# Graphene–Gold Nanoparticles Hybrid—Synthesis, Functionalization, and Application in a Electrochemical and Surface-Enhanced Raman Scattering Biosensor

**DOI:** 10.3390/ma9060406

**Published:** 2016-05-24

**Authors:** Ibrahim Khalil, Nurhidayatullaili Muhd Julkapli, Wageeh A. Yehye, Wan Jefrey Basirun, Suresh K. Bhargava

**Affiliations:** 1 Institute of Postgraduate Studies Building, Nanotechnology & Catalysis Research Centre (NANOCAT), University of Malaya, Kuala Lumpur 50603, Malaysia; ikhalilcu@gmail.com (I.K.); nurhidayatullaili@um.edu.my (N.M.J.); 2 Institute of Postgraduate Studies, Department of Chemistry, University of Malaya, Kuala Lumpur 50603, Malaysia; jeff@um.edu.my; 3 Nanotechnology & Catalysis Research Centre, University of Malaya, Kuala Lumpur 50603, Malaysia; 4 Centre for Advanced Materials & Industrial Chemistry (CAMIC), School of Applied Sciences, RMIT University, GPO Box 2476, Melbourne 3001, Australia

**Keywords:** graphene, graphene–gold nanoparticle, electrochemical biosensor, SERS biosensor, bioimaging

## Abstract

Graphene is a single-atom-thick two-dimensional carbon nanosheet with outstanding chemical, electrical, material, optical, and physical properties due to its large surface area, high electron mobility, thermal conductivity, and stability. These extraordinary features of graphene make it a key component for different applications in the biosensing and imaging arena. However, the use of graphene alone is correlated with certain limitations, such as irreversible self-agglomerations, less colloidal stability, poor reliability/repeatability, and non-specificity. The addition of gold nanostructures (AuNS) with graphene produces the graphene–AuNS hybrid nanocomposite which minimizes the limitations as well as providing additional synergistic properties, that is, higher effective surface area, catalytic activity, electrical conductivity, water solubility, and biocompatibility. This review focuses on the fundamental features of graphene, the multidimensional synthesis, and multipurpose applications of graphene–Au nanocomposites. The paper highlights the graphene–gold nanoparticle (AuNP) as the platform substrate for the fabrication of electrochemical and surface-enhanced Raman scattering (SERS)-based biosensors in diverse applications as well as SERS-directed bio-imaging, which is considered as an emerging sector for monitoring stem cell differentiation, and detection and treatment of cancer.

## 1. Introduction

The advent of graphene, a perfect two dimensional (2D) material, composed of single-atom-thick sheets of sp^2^ bonded carbon atoms packed into a honeycomb lattice, has opened up the exciting new horizon of the carbon era in the field of science and technology. From its discovery in 2004 by Geim and Novoselov [1], graphene has attracted increasing attention due to its excellent properties and applications in diversified fields [2,3]. Owing to its structural features, graphene is characterized by a number of unique and extraordinary structural, optical, and electronic properties (Table 1) [4] with mesmerizing transport phenomena such as the quantum Hall effect [5], optical transmittance, and fluorescence quenching ability [6]. Graphene is a zero-band-gap semiconductor and demonstrates high electron mobility under ambient conditions, [7] which is advantageous in sensors, super capacitors, and electrocatalysis application. The high optical transparency of graphene nanocomposites pushes forward the fabrication of transparent conductive films [8,9] for application in solar cells, advanced electronics *etc*. All of these properties make graphene an ideal building block in the fabrication of nanocomposites. Graphene nanocomposites also show high thermal conductivity that provides excellent thermal stability, which is important in some electronic devices or catalytic reactions that release heat, such as fuel cells and lithium-ion batteries.

**Figure 1:**
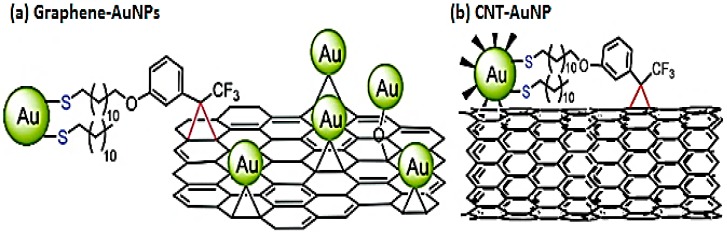
(**a**) Decoration of AuNPs on graphene. Adapted from [23], with permission from ©2011 American Chemical Society; (**b**) Covalent attachment of AuNP on CNT. Adapted from [24], with permission from ©2011 American Chemical Society.

Before graphene, another carbon nanomaterial, carbon nanotubes (CNTs), were of great interest in the fabrication of nanocomposites in biosensor applications [16,17], however, the preference for this material seems to have declined with the emergence of graphene due to its easy availability and some other advantageous properties in comparison to CNTs [18]. Graphene has a unique basal plane structure to load microspheres of several hundred nanometers in diameter, which presents a benefit over CNTs for nanomaterial decoration (Figure 1) [19]. Its 2D structures make it plausible to synthesize graphene-based nanocomposites by novel synthesis methods such as thermal decomposition of intercalated graphene precursors, which is a challenge in the case of CNT-based nanocomposites [20,21]. The higher surface area of graphene improves interfacial contact with the other components in comparison to CNTs and can prevent the accumulation of secondary components, thus preserving some unique properties in the nanoscale level [22]. In addition, graphene has no metallic impurity, which is the major drawback of CNTs in biosensor applications, and hence can be easily integrated into complex sensors or other devices through conventional microfabrication approaches. Conversely, the one-dimensional nature of CNTs creates difficulty in controllably assembling integrated electronic architectures on them.

Nanocomposites consist of multiphase materials wherein one phase (dispersed phase) in nanosize form is dispersed in a second phase (matrix/continuous phase), with the ensuing combination of the individual properties of the component materials [25]. Graphene–inorganic metal and metal oxide nanocomposites are now substrates of interest due to their advantageous properties in diversified fields of application. In some instances, these composites not only overcome the limitations of the usage of a single component in biosensor applications but also provide higher effective surface area, excellent catalytic properties, higher specificity, limit of detection (LOD), *etc.* For example, individual sheets of graphene have a tendency towards irreversible self-agglomerations [26] by van der Waals and π-π stacking interactions, which may partially reduce their electrochemical properties. The addition of a second component (noble metal nanoparticles) acts as a nano-spacer and conductor, hence increasing the graphene interlayer distance to minimize the agglomeration, making both faces accessible and improving the electrical conductivity [27,28].

Direct immobilization of biomolecules (proteins) onto CNTs [29] or graphene oxide (GO) [30] has been proved unstable, therefore frequently applied washing steps in biosensor fabrication can readily remove proteins. Consequently, this presents undesirable effects, such as poor reliability/repeatability and non-specificity of the sensor. Graphene–nanoparticle hybrid structures offer a number of highly desirable and markedly advantageous additional unique physicochemical properties and functions in bio-applications in comparison to either material alone [31]. Among the noble metal nanoparticles, AuNPs are one of the most studied nanomaterials, due to their remarkable surface chemical properties [32], higher chemical stability, excellent catalytic activity [33], biocompatibility [34], and other notable properties. These properties make AuNPs a model component for the detection of DNA [35,36,37] and proteins [38], rapid identification of microorganisms [39], and differentiation of cancer patients from healthy individuals [40].

Therefore, it is highly expected that extraordinary outputs can be achieved using the fabricated graphene–AuNPs composites in numerous applications. In this regard, AuNPs/reduced graphene oxide (rGO) composites comply by offering around 2.3 times superior electrocatalytic current density [41], and stronger Raman signals from Rhodamine 6G (R6G) absorbed on the nano-composites than individual pure AuNPs [42]. In addition, the presence of Au and Ag nanostructures (AgNS) on graphene increases the SERS by factors of about 45 and 150, respectively, than graphene alone [43]. This review emphasizes the wide-ranging synthesis and fabrication procedures of graphene–AuNPs hybrids, their application as a fundamental component for the electrochemical and SERS-based biosensor, as well as SERS-measured bioimaging.

## 2. Fabrication of Graphene–Gold Nanocomposite

**Figure 2:**
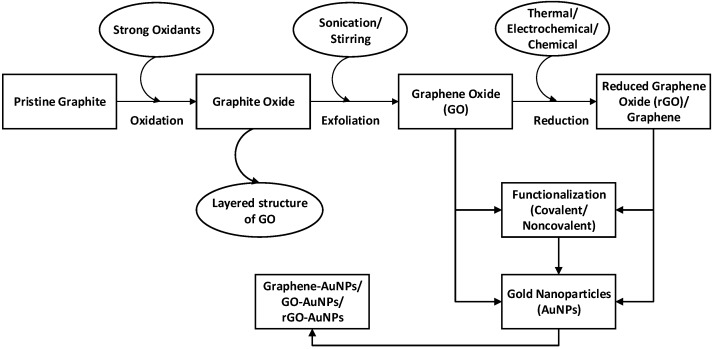
Schematic representation of the formation of graphene–AuNPs nanocomposites.

Considering the unique and advantageous properties of GO and its derivatives—graphene and rGO, efforts have been made to utilize these materials either by themselves, or in conjunction with other nanomaterials. On the basis of structural features, graphene nanoparticles can be broadly categorized into two main classes: AuNPs-embedded graphene nanocomposites and graphene-encapsulated AuNPs. This section introduces the methods used to produce graphene, GO, and rGO, and presents an in-depth analysis of the various synthesis methods of graphene–nanoparticle hybrids with particular emphasis on graphene–AuNPs. A schematic representation of the synthesis of graphene–AuNPs composites is drawn in Figure 2.

### 2.1. Synthesis and Functionalization of Graphene

**Figure 3:**
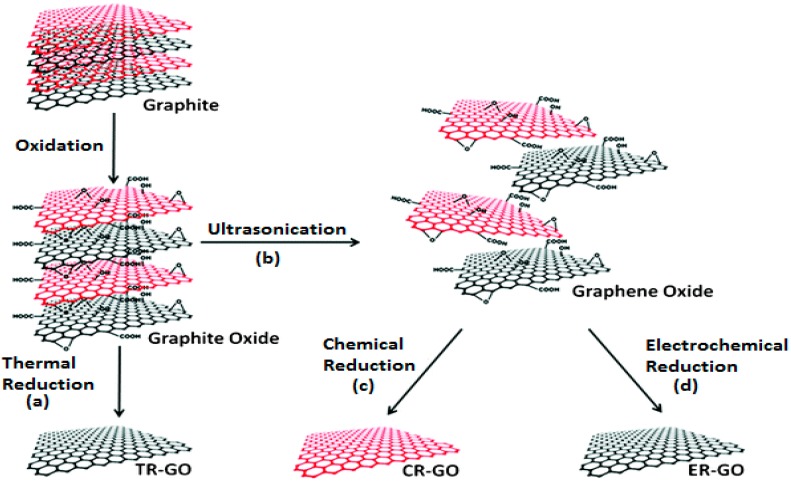
Schematic representation of the synthesis of chemically modified graphene. Adapted from [63], with permission from ©2012 Royal Society of Chemistry.

Graphite oxide, formerly called graphitic oxide or graphitic acid, is the layered structure of GO sheets obtained by treating pristine graphite with strong oxidizers. Chemically, graphite oxide is similar to GO while very different structurally. The material is exfoliated into monolayers or a few layered sheets retaining a rather stacked structure [44] (Figure 3). This exfoliation to colloidal suspension of GO sheets in water or organic solvents is generally mediated by simple sonication [45,46] and by stirring for a longer period of time [47]. Graphite oxide and GO are electrically insulating materials due to their interrupted sp^2^-bonding networks, hence conductivity can be regained by rearranging the π-network by the reduction of GO. The product of this reaction is termed differently either as graphene, rGO, or chemically reduced graphene oxide (CR–GO). rGO is the most common product among the synthesized chemically modified graphene which is generally attained by graphite oxide exfoliation–chemical reduction pathway [48]. Nowadays, most graphene-based nanocomposites are considered desired hybrid materials, employing graphite oxide as the initial material. Till now, a lot of articles as well as reviews have been published on the different synthesis techniques of graphene, highlighting different properties including dimensions, layers, conductivity, quality, cost effectiveness, and so on [49,50,51,52]. Methods include micromechanical exfoliation [1], chemical vapor deposition (CVD) and epitaxial growth [53,54], epitaxial growth on electrically insulating surfaces [55,56,57], colloidal suspension from graphite or graphite derivatives [46,58], longitudinal “unzipping” of CNTs [59,60], and chemical, electrochemical, or thermal reduction of GO [26,61,62]. Here, we present a comparative study (Table 2) of the methods that are scalable as well as mostly employed in research and bio-applications.

Graphene nanosheets can be functionalized, in some instances, to attain high specificity, greater loading capacity, solubility, stability, and biocompatibility [64]. Generally, this can be achieved by either covalent bonding of the functional molecules between the basal planes and edges of GNs or noncovalent adsorption via hydrogen bonding [65], π-π stacking [66], electrostatic interactions, and van der Waals attractions [67]. The expected property of graphene nanosheets (GNs) can be attained by creating covalent hydroxyl (-OH) or carboxyl (-COOH) groups, treating with strong acid/oxidants, sulfonate (-SO3, -SO3H) and amino groups on the graphene surface, immobilizing linker molecules such as pyrenebutyric acid and molecules with an aromatic tail or a reactive end [64], and by adding polymers [68,69] or small molecules [70,71]. Thus functionalization turns graphene/GO into a versatile precursor for a wide range of executions, such as boosting the solubility of graphene in various solvents [71,72], augmenting the capability to adhere to nanomaterials or disperse in matrices [73], and improving the manipulation and processing aptitude of graphene for the fabrication of different devices [74].

### 2.2. Synthesis and Functionalization of Graphene–Gold Nanoparticles

**Figure 4:**
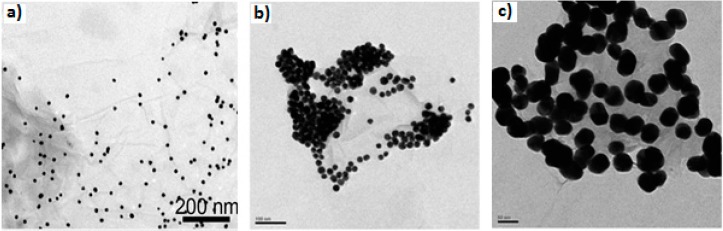
TEM image of GO–AuNPs composites (**a**) *in situ* growth, adapted from [81], with permission from ©2014 Nature Publishing Groupand (**b**) and (**c**) *ex situ* decoration of 20 nm and 40 nm AuNPs on GO sheets respectively, adapted from [82], with permission from ©2010 Royal Society of Chemistry.

**Figure 5:**
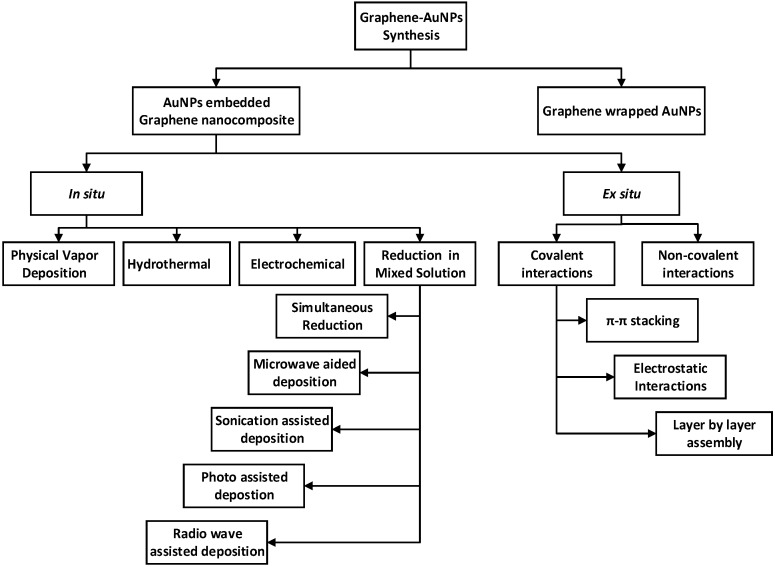
Schematic diagram of the graphene–AuNPs synthesis procedures.

In a broad sense, the synthesis of graphene–AuNPs hybrids can be categorized into two basic categories. The principal approach entitled the *in situ* technique (Figure 4a) involves the formation of nano crystallites in the presence of pristine or functionalized GNs followed by the direct growth of nanostructures onto the graphene surfaces; while the other technique, termed *ex situ*, (Figure 4b,c) comprises the preceding synthesis of nanomaterials in the desired sizes and shape, followed by modification and subsequent attachment to the surface of functionalized GNs [25,76]. Under these two broad titles, there are many different synthesis techniques which are illustrated in Figure 5. Furthermore, beneficial aspects as well as limitations of the major synthesis procedures of graphene–Au nanocomposites are summarized in Table 3.

#### 2.2.1. *In situ* Synthesis of Graphene–Gold Nanoparticles

##### Simultaneous Reduction

The common path of graphene–AuNPs synthesis is the synchronous reduction of Au metal precursors and GO in a mixed solution. The fundamental principle is that the functionalities on GO or rGO surfaces cause the attachment of free metal ions through electrostatic interactions while the addition of a reducing agent expedites the coupling of metal ions. The spontaneous reduction of Au ions in the absence of any reducing agent and linker molecule to form layer-by-layer (LBL) films of alternating graphene and AuNPs has also been shown. This simple and cost-effective method produces more electrically conductive rGO than GO sheets [93]. In another method, both AuNP precursors and GO are reduced individually with the addition of different reducing agents [94]. The functionalization of GO or its derivatives has also been observed by adding external molecules for better output [69], dispersion, size distribution, or even stability of the AuNPs [95,96]. Commonly used functionalizing agents include—octadecylamine (ODA) [95], 1-pyrene butyric acid [96], tannic acid [97], pyrene ethylene glycol amine or decyl pyrene [69], sulfur [98], and others. On the contrary, however less frequently, functionalization of the AuNPs to accelerate the reduction process has also been observed [83]. Different synthesis approaches along with different reducing, functionalizing, and stabilizing agents or conditions are summarized in Table 4.

##### Microwave-Assisted Deposition

Microwave irradiation triggers the uniform and prompt heating of reaction mixtures, thereby allowing simultaneous reduction of GO and metal ions, resulting in the rapid formation of Gr–AuNS. Therefore, nanoparticles of very little size with narrow size distribution can be achieved [99]. For instance, Hu *et al.* [100] reported a GNs–Au nanocomposite by microwave irradiation and Jasuja *et al.* [101] evidenced *in situ* synthesis of multiple shaped bare-surfaced AuNPs on GO sheets by applying microwave exposure without any reducing agents and stabilizing molecules. Simultaneous chemical reduction of GO to rGO with the formation of AuNPs nanosheets in presence of ascorbic acid [102] and prior functionalized GNs with polyethyleneimine and HAuCl_4_ to prepare Gr-AuNPs [103] were also reported. Microwave exposure strength as well as other parameters are mentioned in Table 4.

##### Sonication-Assisted Deposition

High-frequency ultrasound reduction methods are being considered as an expedient and clean approach for the synthesis of Gr–AuNPs nanocomposites. For example, Park *et al.* reported the synthesis of rGO–AuNPs nanocomposites by simultaneous reduction and deposition of AuNPs onto the surface of rGO by ultrasonic irradiation. Here, the attachment of AuNPs onto the rGO surface is mediated via the electrostatic attraction of Au ions to oxygen functionalities on the rGO surface [104]. On the other hand, Vinodgopal *et al.* [105] reported simultaneous and sequential reduction of GO and HAuCl_4_ in 2% polyethylene glycol aqueous solution to fabricate rGO–Au nanocomposites by maintaining an ultrasonic frequency of 211 kHz.

##### Photo-Assisted Deposition

Photo-assisted deposition is a green technique which creates a uniform reducing environment in solution without the need for any additional reagents. For example, GO–gold nanorods (AuNRs) synthesis by the one-pot one-step method was achieved by UV light irradiation (256 nm, 30 W) for 25 min in a quartz tube [106]. Huang *et al.* [107] reported one-pot synthesis of Au nanodots (AuNDs) on rGO nanosheets by photochemical reduction of HAuCl_4_ in the presence of octadecanethiol. Another group of scientists prepared ultrathin gold nanocrystals (AuNCs) on a co-reduced GO surface by photo irradiation (LEDs with continuous stirring) in the absence of chemical reductants and surfactants [108]. Also, photo-aided synthesis of graphene–AuNPs was employed to grow AuNPs in AuCl_4_^−^ electrolyte [109]. This convenient and reliable method has ensured a steady growth rate of AuNPs with well-controlled distribution by using focused laser light.

##### Radio Wave-Assisted Deposition

Pruneanu *et al.* [110] employed the radio frequency (1.2 MHz, 5 kW) CVD method to synthesize multi-layered graphene–AuNPs composite by using Au/MgO catalyst (1 wt % Au).

#### 2.2.2. Electrochemical Method

The electrochemical reduction method is a simple, fast, and green technique of graphene–AuNPs fabrication. The classical electrochemical deposition method consists of three steps: firstly, deposition of graphene sheets onto an electrode, secondly, immersion of the graphene-coated electrode in an electrolytic solution containing metallic precursors, and finally application of an electrochemical potential. Generally, glassy carbon electrode (GCE) as a working electrode material and an *in situ* technique is mainly applied for the electrochemical deposition of graphene–AuNPs. Table 5 summarizes different reduction methods, electrochemical potentials, and reaction conditions for the fabrication of graphene–AuNPs composites.

#### 2.2.3. Hydrothermal Reduction Method

Usually, the hydrothermal reduction method is performed at high temperature with high steam pressure using an autoclave. The most advantageous feature of this approach is the formation of nanoparticles or nanowires without any requirement for post annealing and calcination [25]. Qin *et al.* [85] reported the fabrication of GO–AuNCs (80% of pentagonal pyramid and 20% of tetrahedron) of 10–20 nm in size variation through a hydrothermal reduction and crystallization route using polytetrafluoroethylene autoclave at 60 °C overnight without using extra reductants or capping agents. Liu *et al.* [87] described the preparation of graphene–AuNPs in a Teflon-lined autoclave with a microwave hydrothermal system at 150 °C for 60 min in the absence of reducing agents.

#### 2.2.4. Physical Vapor Deposition Method

This procedure ensures the formation of metal nanoparticles of different and controllable geometries on the graphene sheets. Zaretski *et al.* [127] deposited metal nanoislands on a metal/graphene bilyer template by keeping the evaporation rate at 0.1 Å·s^−1^ and chamber pressure at 7 × 10^−7^ torr, whereas Pandey *et al.* [128] fabricated AuNPs on graphene with appropriate control over size (down to ~1.5 nm) and coverage (5 × 10^4^ μm^−2^). In physical vapor deposition, the size of the gold atoms deposited onto the graphene depends on the number of the layers [127,128,129]. Besides, the geometry, distribution, and inter-island gaps of the gold metal deposited on graphene rely on the type of the substrate materials, and reaction conditions [127,128]; the morphology varies significantly even on the type of the metal itself [128].

#### 2.2.5. *Ex Situ* Method

In the *ex situ* approach, nanoparticles are synthesized in advance and subsequently decorated onto the surface of graphene sheets. This attachment is facilitated by either covalent or noncovalent interactions, including van der Waals interactions, hydrogen bonding, π-π stacking, or electrostatic interactions. In this method, metal nanoparticles or graphene, or sometimes both, necessitate activation with functional groups [130]. However, the type of functionalization and interaction strength defines the loading, *i.e.*, dispersion and concentration of metal nanoparticles on the graphene surface [131]. Therefore, the *ex situ* self-assembly procedure is a promising technique to overcome the difficulties encountered during the *in situ* technique for nanocomposite fabrication [25].

##### Covalent Interactions

The *ex situ* approach for noble metal nanoparticles decoration is not often used. GO rather than rGO is preferable for covalent attachment of nanoparticles due to the vast amount of oxygen functionalities on its surface which facilitate bonding with other functional groups. Ismaili and co-workers demonstrated the light-activated (wavelengths above 300 nm) covalent formation of 3-aryl-3-(trifluoromethyl)-diazirine modified AuNPs on rGO [23].

##### Noncovalent Interactions

- π-π stacking

**Figure 6:**
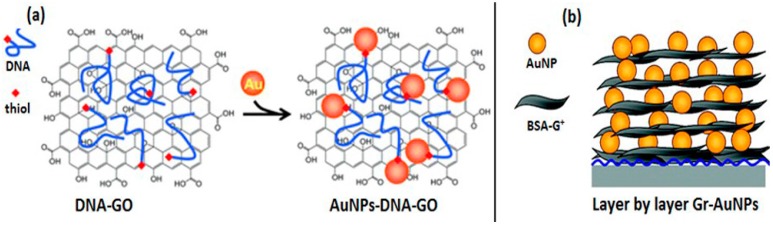
*Ex situ* Graphene-–AuNPs decoration (**a**) noncovalent interactions, adapted from [132], with permission from ©2009 Royal Society of Chemistry; (**b**) LBL self-assembly, adapted from [118], with permission from ©2012 American Chemical Society.

Generally, aromatic compounds are attached to the nanoparticle surface, which enables their attachment to graphene via π-π stacking. For example, pyridine, purine, and pyrimidine bases of DNA [132], thiolated DNA (Figure 6a) [132], and pyrene-labeled DNAs [133] have provided π-π interactions between AuNPs and GO/rGO/graphene sheets. Guided by similar principles, Wang *et al.* fabricated DNA conjugated AuNPs and AgNPs on GO nanosheets, respectively, by functionalizing nanoparticles with DNA via didentate capping ligands, followed by assembly onto GO via π-π stacking interactions [134].

- Electrostatic interactions

This is one of the most common as well as being considered a facile and scalable method of synthesizing graphene–metal nanoparticles in a precise manner avoiding conglomeration. The common principle behind this reaction is that GO and rGO have immanent negative charges on their surface which are being utilized to assemble/decorate positively charged AuNPs. For instance, Mao *et al.* [96] have described the electrostatic force-directed assembly of AuNP–antibody conjugates onto the surface of TR-GO sheets.

- Layer-by-layer self-assembly

This bilayer film is typically generated by alternating the oppositely charged GNs and AuNPs. Consecutive repetition of the decoration process is employed to make the desired number of bilayers. Liu *et al.* fabricated graphene/AuNPs multilayered films consisting of 4-styrenesulfonate functionalized rGO and polyamidoamine dendrimer stabilized AuNPs on GCE adapted with an initial layer of poly(diallyldimethylammonium chloride) [135]. Xi *et al.* [118] reported a uniform three dimensional (3D) AuNPs-inserted graphene thin film by the electrostatic LBL assembly of AuNPs and bovine serum albumin-functionalized GNs (Figure 6b) followed by thermal annealing at 340 °C for two hours under aerobic conditions (Figure 6b).

#### 2.2.6. Graphene-Wrapped Gold Nanoparticles

**Figure 7:**
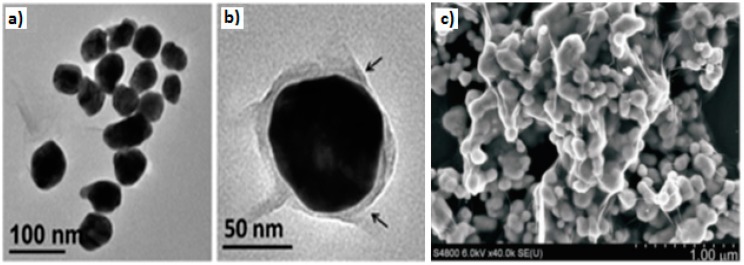
TEM images of the Au-encapsulated GO nanoparticles at (**a**) low magnification; (**b**) high magnification, adapted from [140], with permission from ©2013 Royal Society of Chemistry and (**c**) SEM image of GO-wrapped AuNPs, adapted from [141], with permission from ©2014, 2015 Wiley.

Graphene, GO, or rGO can be easily used to wrap or encapsulate the AuNPs with variable sizes ranging from nanometer to even micrometer level due to their flexibility and 2D nature [136]. The oxygen functionalities on GO and rGO generate an overall negative zeta potential, thus easing coupling with positively charged AuNPs. This encapsulation process enhances the greater degree of contact, results in the suppression of AuNPs aggregation, and ensures greater stability, thereby limiting the degree of exfoliation of AuNPs from graphene [76]. One of the initial efforts is the fabrication of a graphite-like carbon shell around the AuNPs attached to a 3-mercaptopropyl-trimethoxysilane-modified Si substrate, followed by the growth of a graphene shell using the CVD process [137]. Bian *et al.* [138] applied the CVD method for growing graphene shells onto the loaded HAuCl_4_ metal precursors on fumed silica powder, followed by silica dissolution to retain the graphene-encapsulated AuNC. On the other hand, Kim and co-workers [139] achieved the desired structure by using aminopropyltriethoxysilane-functionalized ITO to decorate AuNPs and followed by encapsulation by the GO via electrostatic interaction. GO-wrapped AuNPs hybrid materials are now constructed without using any substrate, which ensures consistent wrapping of GO sheets onto each of the AuNPs (Figure 7a,b) [140] as well as controllable, tunable size and morphology of the AuNPs (Figure 7c) [141].

**Figure 8:**
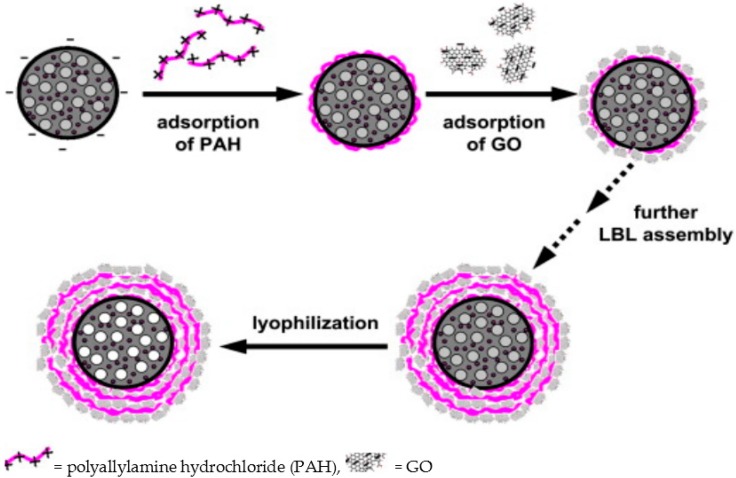
LBL fabrication process of Au@PLA–(PAH/GO)*_n_* microcapsule. Adapted from [148], with permission from ©2013 Elsevier.

As per the requirement of specific features, particular approaches are executed: electrostatic self-assembly of ultrathin GO-wrapped AuNPs or AuNRs with excellent dispersibility of individual nanoparticles [142], *in situ* reduction of electrostatically bounded nanosized GO to cysteamine-stabilized AuNPs or Cetyl-trimethylammonium bromide (CTAB)-stabilized AuNRs accompanied by greater colloidal stability as well as enhanced photothermal effect [143]. Turcheniuk *et al.* [144] reported pegylated (PEG) rGO nanoparticles and AuNRs coated with rGO–PEG (rGO–PEG–AuNRs) by laser irradiation to achieve the declined cytotoxicity of the CTAB-stabilized AuNRs and enhanced overall photothermal activity. It is observed that the encapsulation of AuNS by GNs is generally accomplished by electrostatic self-interactions. In some cases, firm bonding is needed along with expected properties and necessitates different strategies such as functionalization of the AuNPs [145], generation of different shapes, combinations, and arrangements of AuNS [146,147], and even by LBL self-assembly [148]. Jin *et al.* [148] fabricated a complex architecture by introducing AuNPs into polylactic acid (PLA) microcapsules through a dual microemulsion water-in-oil-in-water solvent evaporation technique followed by electrostatic LBL deposition of GO on the microcapsule surface (Figure 8).

## 3. Graphene–Gold Nanoparticle Hybrid for Biosensing and Bioimaging Application

Biosensors are self-contained analytical devices using biological sensing elements that respond selectively and reversibly to detect and/or quantify a particular target analyte or family of analytes. A biosensor is made up of two fundamental components—(i) receptor and (ii) transducer. The receptor may be of either organic or inorganic material which interacts with a marked analyte or group of analytes. Conversely, a transducer transforms the recognition event which occurred between the analyte and the receptor into an assessable signal. These signals can occur in varied forms including, but not restricted to, electrical, electrochemical, and optical.

Nanoparticles can be used as biosensor platforms to enhance sensitivity by amplifying the obtained signal as well as increasing the available surface area for analyte binding. Graphene–AuNPs hybrids made up of two excellent and unique modalities in this context ensure a number of advantageous properties in biosensing applications. Graphene itself is an excellent material with which to immobilize nanoparticles, enhancing their stability, e.g., preventing aggregation while graphene with nanoparticles increase the available surface area for analyte binding, as well as improving their electrical conductivity and electron mobility, thereby enhancing the achievable sensitivity and selectivity. In the next section, we focus primarily on the use of graphene–AuNP hybrid materials in electrochemical and optical biosensors with greater emphasis on how they compare to current gold standards and their sensitivities and selectivities towards various biomolecules.

### 3.1. Electrochemical Biosensor

Electrochemical biosensors are, by far, the largest group of sensors which provide especially attractive methods of analyzing the content of a biological sample due to direct conversion of a biological recognition event to an electrical signal. A typical electrochemical biosensor consists of a sensing (or working) electrode containing a biological recognition element and a counter electrode, both detached by a layer of electrolytes. At electrochemical biosensing, in most cases silver/silver chloride (Ag/AgCl) and platinum (Pt) wire/sheets are being used as the reference and auxiliary/counter electrodes respectively, against different graphene–AuNPs-based working electrodes. On the basis of the nature of their biological recognition process, electrochemical biosensors can be categorized into—(i) affinity sensors; and (ii) catalytic devices [149]. Affinity-based sensors depend on the selective binding properties between a biological element such as an antibody, enzyme, nucleic acid, or a receptor and its target analyte, which results in the production of a measurable electrical signal. On the other hand, catalytic sensors generally incorporate nanoparticles or enzymes, whole cells, or tissue pieces that identify the target analyte and yield electroactive species [149]. Various forms of voltammetry/amperometry (e.g., linear sweep, differential pulse, square wave, stripping), impedimetry, and potentiometry techniques are commonly used for the electrochemical detection of biomolecules [150,151]. The amount of analyte being reduced or oxidized at the working electrode is proportional to the concentration of the target analyte present. In this context, graphene is being considered as a perfect conductor of electrical charge material [152,153]. Furthermore, the high surface area of graphene facilitates the formation of a large number of defects and, consequently, electroactive sites, due to the heterogeneous electron transfers that can occur between graphene and the analyte to be oxidized or reduced [154]. The electrochemical behavior of graphene is first-rate and analogous to other carbon-based materials, including CNTs and graphite. Current researches have shown that graphene-based electrochemical biosensors exhibit better performance than CNTs due to the presence ofmore sp^2^-like planes and surface edge defects. While graphene exhibits a great prospective, graphene nanoparticle composites, including metal nanoparticles, metal oxide, and semiconductor nanoparticles, have recently paved more attention toward their electrochemical sensing capability [155]. These nanoparticles exhibit different roles, for example, the immobilization of biomolecules, catalysis of electrochemical reactions, or acting as a reactant in electrochemical sensing platforms [153,156,157,158].

In the biomedical field, electrochemical biosensors are currently showing the dominating trend. The fabrication of electrochemical biosensors using graphene–AuNPs for glucose sensing is one of the prime and mostly applied methods. For example, Shan *et al.* [159] constructed a novel and biocompatible graphene/AuNPs/chitosan nanocomposite on an Au electrode which shows high electrocatalytical activity toward H_2_O_2_ and O_2_ due to attributes of the synergistic effects between graphene and AuNPs. Influenced by this result, a glucose biosensor is manufactured by immobilizing glucose oxidase (GOD) on thin films of graphene/AuNPs/chitosan nanocomposites on an Au electrode. The resulting biosensor reveals a remarkable amperometric response to glucose with good reproducibility and LOD of 180 μM with a liner range of 2–10 mM, which makes it applicable to real-time clinical analysis of blood glucose levels (4–6 mM) [159]. Therefore, different combinations of graphene–Au nanocomposites have been tested for glucose biosensing by immobilization of the GOD enzyme, including AuNPs–rGO/GCE [114], graphene/nano-Au/GOD/GCE [124], AuNPs–graphene/GCE [160], graphene/ polyaniline(PANI)/AuNPs/GCE [161], AuNPs–graphene rod [162] and GCE-4-aminothiphenol(ATP)-GNs–AuNPs [98]. Here, at first a GNs–AuNPs hybrid film is prepared by the wet impregnation-thermal reduction method followed by their deposition on a modified glassy carbon electrode (GCE) via LBL assembly of ATP and GCE for up to three layers. GOD is immobilized and checked for glucose-sensing efficiency both by voltammetry and electrochemical impedance spectroscopy (EIS). EIS experimental data analysis indicates the enhanced activity which might be due to the synergistic effect of GNs and AuNPs, the role of ATP mediating the assembly of the GNs–AuNPs hybrid on GCE, and the increase in surface roughness [98]. LBL deposition of GOD on graphene–AuNPs is also mentioned by another group of scientists [163].

On the other hand, a non-enzymatic glucose voltammetric sensor developed by Ruiyi *et al.* [119] based on GA@AuNPs/AuNPs shows ultra-high sensitive electrochemical response to glucose due to greater electron transfer, mass transport, and catalytic activity. The study of the prepared GA@AuNPs/AuNPs shows high electrical conductivity (15.4 S·m^−1^), specific surface area (291.6 m^2^·g^−1^), and an apparent heterogeneous electron transfer rate constant (14.8 ± 0.12 cm·s^−1^) because of their well-exposed active sites as well as the high catalytic properties of the adsorbed AuNPs. The enzyme-free voltammetric glucose sensor based on graphite/SrPdO_3_ perovskite/AuNPs nanocomposites offers many advantages for glucose electro-oxidation such as high sensitivity, low detection limit, and excellent long term stability [164]. It has shown high selectivity to glucose even in presence of common interferences like ascorbic acid, uric acid, paracetamol, dopamine, and chloride. Furthermore, it is also proved to be an excellent sensor for glucose sensing in human urine and blood serum samples with outstanding recovery and low LOD of 16.55 and 14.25 µmol·L^−1^, respectively [164].

**Figure 9:**
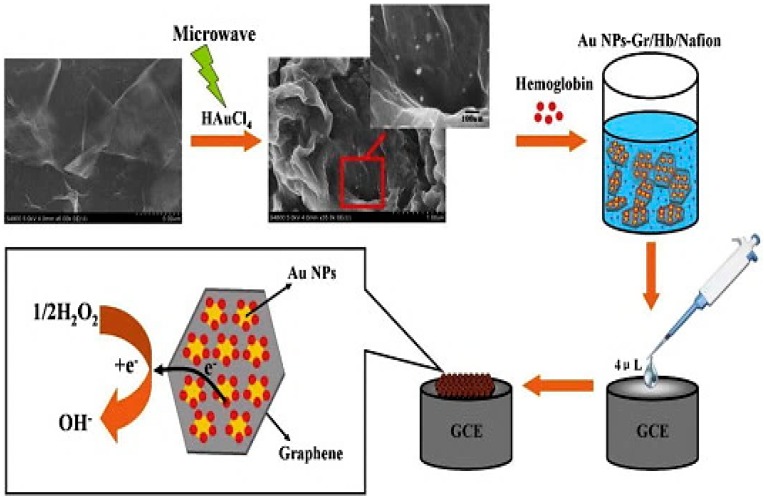
Fabrication steps of AuNPs–Graphene/Hb/Nafion/GC electrode and electrocatalytic activity for H_2_O_2_. Adapted from [87], with permission from ©2014 Elsevier.

Another good example is the utilization of AuNPs-decorated graphene nanocomposites in the catalysis of electrochemical reactions to detect H_2_O_2_. For example, Fang *et al.* [165] fabricated a graphene–AuNPs hetero-structure using poly(diallyldimethylammonium chloride)-functionalized graphene which ensured high loading and uniform dispersion of AuNPs on the GNs, as well as making the sensor an efficient one with low LOD and a wide linear range compared to that of an enzyme-based sensor. On the other hand, graphene–AuNPs immobilized with hemoglobin to construct the Nafion/hemoglobin (Hb)/AuNPs–graphene/GCE sensors show ultra-sensitivity for H_2_O_2_ detection (LOD 0.03 μM) with good reproducibility and longer stability (Figure 9) [87]. Chang and co-workers [166] used LBL-assembled AuNP–graphene–poly (toluidine blue O) hybrid films for the detection of H_2_O_2_ by evaluating the oxidative stress of a tumor cell. The results indicated a higher efflux of H_2_O_2_ in tumor cells compared to normal cells. Similarly, the excellent sensing features of the graphene–AuNPs composite coupled to electrochemical mechanisms have influenced the detection of several other biomolecules and imparted a greater emphasis on biomedical applications, namely, the detection of uric acid [96] and β-nicotinamide adenine dinucleotide (NADH) [41] in human urine with effective separation from the common interferents (glutathione, glucose, ascorbic acid, and quinine), and many others.

**Figure 10:**
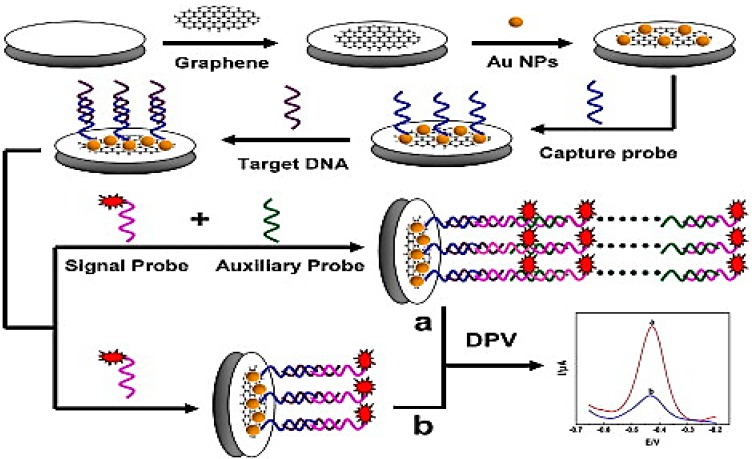
Schematic representation of the fabrication procedure of the DNA biosensor. (**a**) DPV cures from the super-sandwich biosensor; (**b**) DPV cures from the sandwich biosensor. Adapted from [167], with permission from ©2015 Elsevier.

Fabrication of electrochemical biosensors using graphene–AuNP hybrids for DNA detection is one of the most advisable methods nowadays. Wang *et al.* [125] showed that nano electrode ensembles (GCE–GO–AuNP) can be easily modified by thiolated probe DNA (HS-DNA) through strong Au–S bonding. Addition of the target DNA or 1-mismatch target DNA (m-DNA) facilitates the hybridization of probe DNA with intercalation of methylene blue into the DNA duplex, specifically by binding with guanine in DNA molecules. A super-sandwich type electrochemical biosensor (Figure 10) was fabricated by Wang *et al.* [167] using a methylene blue-labelled signal probe for sequence-specific DNA detection with ultra-sensitivity and single-base mismatched target DNA detection. Conversely, Peng *et al.* [168] constructed AuNPs/toluidine blue–GO-based (AuNPs/TB–GO) label-free biosensor for the detection of the multidrug resistant 1 (MDR1) gene responsible for the resistance to chemotherapeutic drugs used in the treatment of human cancer. The developed sensor showed very low LOD with a wide linear range as well as an ability to differentiate between single-base mismatched DNA sequences among the MDR1-related DNA sequences. Sun *et al.* [89] reported an electrochemical DNA biosensor made up of multilayer graphene–AuNPs immobilized with a dual-labelled (50-SH and 30-biotin) stem-loop DNA probe. This DNA biosensor is extremely effective in the detection of the peanut allergen-Ara h1 gene from peanut milk beverages as well as highly sensitive and selective to the target DNA sequence with great recovery (86.8%–110.4%).

**Figure 11:**
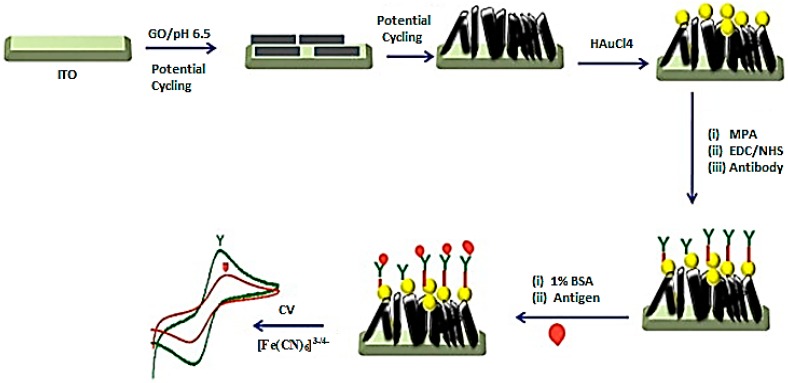
Fabrication of eGr–AuNP on ITO for immune sensing of estradiol. Adapted from [120], with permission from ©2013 Elsevier.

Some pathogenic bacterial species are very difficult to isolate and identify due to their low growth rate and fastidious nature. Hence, their rapid and sensitive detection are crucial to laboratory diagnosis and appropriate patient management. *Mycobacterium tuberculosis* is one of the most problematic bacteria of worldwide public health concern. Tuberculosis is easily transmissible via air, hard to isolate, and has spread across borders and developed multidrug resistance. Hence, the fabrication of an electrochemical DNA biosensor to identify *Mycobacterium tuberculosis* is a pressing need to public health and society. Liu *et al.* immobilized a capture probe (specific sequence of the IS6110 gene) on rGO–AuNPs as a sensing platform and a probe-label (AuNPs–PANI) as a tracer label for amplification. The sensor exhibits ultra-sensitive detection of *M. tuberculosis* DNA as low as femto mole (fm) level [169]. Wang *et al.* [170] focused on the preparation of a low cost, robust, rapid, and sensitive impedimetric immunosensor, which is made of anti-*E. coli* O157:H7 antibodies immobilized on an AuNPs-modified free-standing rGO paper electrode (rGOPE) via the biotin–streptavidin system to detect the most prevalent food-borne disease-producing bacteria, *Escherichia coli* O157:H7. Dharuman *et al.* [120] reported an anti-estradiol antibody immobilized eGr–AuNP composite on an ITO surface for the immune sensing of the breast cancer-inducing hormone 17β-estradiol (E2) in the presence of [Fe(CN)6]^3−/4−^. The lowest LOD of the sensor is 0.1 fM, which indicates the viability of the sensor in real life as blood and urine samples of post-menopausal breast cancer patients normally contain 17*β*-estradiol at the picomole level (Figure 11) [120]. Another immunosensor, named the carcinoembryonic antigen (CEA) immunosensor, was constructed by Yu *et al.* [171] for the rapid and sensitive immunoassay measurement of serum CEA concentration by immobilizing the CEA antibody on AuNPs/poly L-arginine (Arg)/rGO/CILE.

The extraordinarily advantageous properties of graphene–AuNPs make them suitable to different applications, including: the determination of biological compounds—levodopa, uric acid, and folic acid simultaneously [90], and ascorbic acid [81], folic acid [126], dopamine [103,135], and animal growth stimulant; in treatment of estrogen deficiency disorders; in veterinary medicine–diethylstilbestrol (DES) [91]; antibiotics–chloramphenicol [112]; antiepileptic drugs; emerging pollutants in ground and surface water–carbamazepine (CBZ) [110]; environmental pollutants–hydroquinone [100], and so on. The integration of biomolecules in some instances enhances the catalytic properties of the graphene–AuNPs hybrid in electrochemical applications. For example, the immobilization of hemoglobin molecules on an AuNPs–graphene–SDS/BPG hybrid electrode increases the electrocatalytic activity toward nitric oxide [94], while Hb-immobilized AuNPs/graphene with biocompatible chitosan (GACS) (Hb/AuNPs/GACS)-modified GCE is used for the electrochemical detection of nitrite at high sensitivity levels within a wide concentration range and is consequently being envisioned to have promising applications in the monitoring of food safety [172]. Besides, a number of chemicals related to food adulteration are also being detected, e.g., bisphenol A (BPA) in baby bottles [122] and in milk samples by an electrochemical aptasensor [123], and aflatoxin B_1_ in spiked food samples [121]. Graphene–AuNPs-fabricated electrochemical biosensors with their corresponding identified analytes, citing linear range and LOD, are summarized in Table 6.

### 3.2. SERS Biosensor

SERS is a Raman spectroscopic technique combining laser spectroscopy with optical properties of metallic nanostructures. It provides a greatly enhanced Raman signal from a Raman-active analyte adsorbed onto metal nanostructure surfaces [174,175,176,177,178,179]. This enhancement factor strongly relies on the size, shape, and composition of the metallic nanostructure and nature of the molecular analyte [180]. The overall SERS effect is due to two different mechanisms—the electromagnetic enhancement (EME) and chemical enhancement (CE). The EM mechanism is based on the interaction of the transition moment of an adsorbed molecule with the electric field of surface plasmons induced by the incoming light on the metal [181], and is dependent on the presence of rough features on the metal surface [182], independent of the probe molecules. CE is due to the interaction of the adsorbed molecules on the metal surface, mostly from the first layer of the charge–transfer resonance between the adsorbate and the metal [181]. Hence, SERS is being considered as a powerful analytical tool for surface and interfacial analysis as it can unveil the molecular fingerprint information and ultrahigh surface sensitivity [183]. It is one of the best techniques for molecular analysis, with very high sensitivities (nano mole or even pico mole level) [184] and the added capability of detecting single molecules [185,186,187,188].

**Figure 12:**
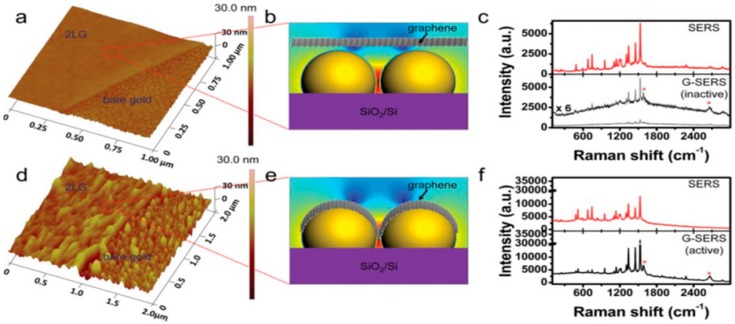
Morphology-dependent SERS performance of normal SERS and graphene-mediated SERS (G-SERS). (**a**,**d**) AFM images of a bilayer graphene (2LG)-covered 8-nm gold film (**a**) before, and (**d**) after annealing, showing both the bare gold regions and graphene-covered regions; (**b**,**e**) Schematic illustration of the contact state between graphene and AuNS correspond to the enlarged regions; (**c**,**f**) SERS performance of normal SERS (top) and G-SERS regions (bottom) (**c**) before, and (**f**) after annealing, respectively. “*” marks the G and G′ band of the 2LG. The figure is adapted from [198], with permission from ©2013 Wiley.

For example, GO-AuNRs have been proved as strong SERS substrates by using a model molecule (cresyl violet perchlorate) that unveiled very large SERS enhancement factors (10^6^) with very low molecular detection limits (10^−11^ M) [106]. It is well known that noble metal nanoparticles (e.g., Cu, Ag, or Au) are more commonly used in SERS-based experiments due to their electromagnetic properties, which enhance the Raman signal [82]. On the other hand, graphene or GO have the potentiality for greater Raman signals via the chemical enhancement mechanism [42,189,190], which is independent from that of noble metal nanoparticles. It can be anticipated that graphene–metal nanocomposites would act synergistically for further magnification of the weak Raman signals by many orders of magnitude via chemical and electromagnetic enhancement when compared to using either graphene or metal nanoparticles alone [82,101,184,191]. Hu *et al.* [192] provided a clear dictation in this context by comparing the Raman signals of an adsorbed aromatic dye molecule, crystal violet onto SiO_2_/Si, GO, Au NRs, and GO-AuNRs separately, and validated the boosted SERS signal of GO-AuNRs nanohybrids. This enhancement is the summation of electromagnetic enhancement based on local electromagnetic field by the AuNRs as the hot spots, and chemical mechanism based on the charge transfer and chemical bonding of GO and crystal violet dye molecules [192]. Zhang *et al.* [193] fabricated SERS-active substrates in a newer dimension based on GO embedded Au@AgNPs sandwich nanostructures (Au@Ag-NPs/GO/Au@Ag-NPs) to achieve higher sensitivity, reproducibility and reliability of the Raman readout and obtained dramatic enhancements of the Raman signals (R6G with an enhancement factor of 7.0 × 10^7^) due to abundant hot spots on their surfaces and the distinctive edifice of the GO sheets. However, a few examples of the SERS-based enhancement are summarized in Table 7. It is experimentally proved that the degree of SERS enhancement could be fine-tuned by the quantity [194], size and shape [191,195,196], type [197] of AuNS on the graphene sheets, and morphological arrangement of graphene and AuNPs (Figure 12) [198], as well as the excitation wavelength of the laser [199]. On the other hand, corresponding enhancement factors are reliant on the volume of graphene and consequentially its thickness [200], layer numbers, *i.e.*, single layer graphene provides larger SERS enhancement in comparison to fewer layers of graphene [199], and type of defects of the graphene sheets. Moreover, in-plane defects in graphene prepared by the CVD technique have defect-enhanced firm interactions of AuNPs with the defect sites and hence a positive influence on the efficient physical functionalization with AuNPs [201]. An experiment by Wang *et al.* [197] proved that ~7 nm thick Au films are the perfect SERS substrates among the different thickness of Au films, decorated on the monolayer graphene for the characterization of low concentration rhodamine molecules, providing the strongest Raman signals for molecules with the weakest photoluminescence background. All of these extraordinary advantageous properties make graphene–Au nanocomposites the perfect substrate for SERS measurements, as it has been extensively instigated in versatile applications, including sensing and molecular diagnostics, biomedical applications, agriculture, food adulteration [202], and so on. This hybrid has also been verified for the detection of single molecule interactions [184], identification of pathogenic microorganisms [203] and biomolecules [204,205], nucleic acids [206], and cancer cells [207], and even in the detection of explosives and chemical warfare agents [208].

Nguyen *et al.* [202] have fabricated a high performance SERS substrate by using graphene–Au films–AuNRs for the detection of three pesticides namely azinphos-methyl, carbaryl, and phosmet by SERS with LODs of approximately 5, 5, and 9 ppm, respectively. The LODs of carbaryl and phosmet meet the FAO/WHO- and EU-defined maximum residue limits, which make it a potential method in food safety applications. On the other hand, Zhang *et al.* [193] developed Au@Ag-NPs/GO/Au@Ag-NPs sandwich nanostructures to detect the pesticide thiram (a broadly applied sulfur-containing pesticide) in commercially marketed grape juice. This hybrid nanostructure shows a narrow detection limit of 0.1 mM (0.03 ppm), which is significantly below the maximal residue limit of 7 ppm in fruit as approved by the United States Environmental Protection Agency. This simple, rapid, and ultrasensitive Raman detection approach shows significant potential in practical applications like on-site monitoring of food/environmental safety and spectroscopic identification of trace pesticides in agricultural foodstuffs [193]. Similarly, Fu *et al.* [117] mentioned GO–AuNPs hybrids as an efficient SERS substrate for the sensitive, selective, and label-free detection of malachite green in water samples, which is a cationic triphenylmethane dye with high genotoxicity and carcinogenicity. Heavy metal contamination is one of the alarming and challenging problems in this 21st century. Mercury (II) (Hg^2+^) is considered one of the most toxic pollutants, having severe adverse effects on the environment and consequently on human health. Therefore, its detection is of prime concern and several approaches have been attempted. In this milieu, Ding *et al.* [209] synthesized heterojunction SERS active substrates, AuNPs/rGO/SiO_2_/Si through *in situ* direct growth of AuNPs on rGO surfaces, which have been utilized for trace analysis of Hg^2+^ via thymine–Hg^2+^–thymine coordination. This heterojunction SERS sensor exhibited 500 times greater enhancement the referenced mercury (II) sensor with an LOD of 0.1 nM or 20 ppt for Hg^2+^.

A glucose biosensor has been manufactured by Gupta *et al.* [210] using immobilized glucose oxidase (GOD) into mercaptophenyl boronic acid (MBA)-terminated Ag@AuNPs–GO nanomaterials films. The developed SERS biosensor shows a linearity range of glucose detection between 2 and 6 mM, with LOD of 0.33 mM, as well as successful determination of glucose in blood samples. This SERS-based analytical method can offer multiple benefits such as selectivity, high speed of analysis, and high cost-effectiveness over other analytical methods. Fan *et al.* [203] demonstrated a popcorn-shaped GO–AuNPs hybrid SERS probe for the ultrasensitive (fM), label-free detection of HIV DNA and the identification of methicillin-resistant *Staphylococcus aureus* in concentrations as low as 10 cfu/mL. He *et al.* [206] fabricated a SERS active platform displaying AuNPs on CVD-made graphene for contemporaneous multiplex DNA examination with a single excitation light source. Here, two different thiolated DNA probes were immobilized on AuNPs, followed by the exposure of target DNA as well as the addition of Cy3-labeled reporter DNA, which resulted in the formation of a sandwich composed of probe/target/reporter DNA. Multiplex detection of DNA was achieved with a LOD of 10 pM. On the other hand, little or no Raman signal was detected from the uncomplimentary DNA at the same concentration. It was also revealed that Raman signals from the Cy3 on AuNPs–graphene/SiO_2_/Si substrate exhibited intense Raman signals compared to the SiO_2_/Si, graphene–SiO_2_/Si, AuNPs–SiO_2_/Si substrates individually, which was attributed to the coupled surface plasmon resonance absorption of AuNPs on the graphene film [206]. The identification of explosive molecules in trace levels is very crucial not only for security screening but also for the environment and human health. Driven by this need, Kanchanapally *et al.* [211] made a GO–Au nanocage hybrid SERS platform for the label-free identification of the nitro explosives cyclotrimethylenetrinitramine (RDX) and trinitrotoluene, with resulting LODs as low as 500 fM and 10 fM, respectively.

### 3.3. SERS Bioimaging

GO–AuNPs hybrids for SERS-based bioimaging have been emerging due to the superior attributes of SERS in this field, such as greater sensitivity, and a stable and reproducible signal over the conventional methods. For example, Ma *et al.* [140] fabricated GO-wrapped AuNPs (AuNPs@nGO) which is employed for intracellular Raman imaging in HeLa cancer cells. It was predicted that AuNPs@nGO enters HeLa cells through endocytosis and is mainly distributed in the cytoplasm. Liu *et al.* [218] found that HeLa cells incubated with GO–AuNPs hybrid exhibited much stronger and more distinguishable Raman signals than the cells incubated with pristine GO. Once again, SERS not only improves the sensitivity but also shortens the acquisition time (1 s). GO–AuNPs are internalized by an energy-dependent process named endocytosis into the subcellular level of individual cells and provide localized sensing and images. The author has provided information on good distribution and stability (several weeks) of the nanohybrid in aqueous dispersion, which indicates higher longevity in intracellular condition. Besides, GO as well as Raman imaging have been approved again as biocompatible and almost harmless to cells, respectively [218]. The internalization events of the hybrid molecules were further supported by a more detailed study conducted by Huang *et al.* [222] by utilizing SERS to illustrate the cellular uptake mechanism of GO–AuNPs nanocomposites in living cells. In their study, Ca Ski cells are considered a model cell line and an inhomogeneous distribution of GO–AuNPs inside the cells is found where internalization is mainly via the clathrin-mediated energy-dependent endocytosis route.

**Figure 13:**
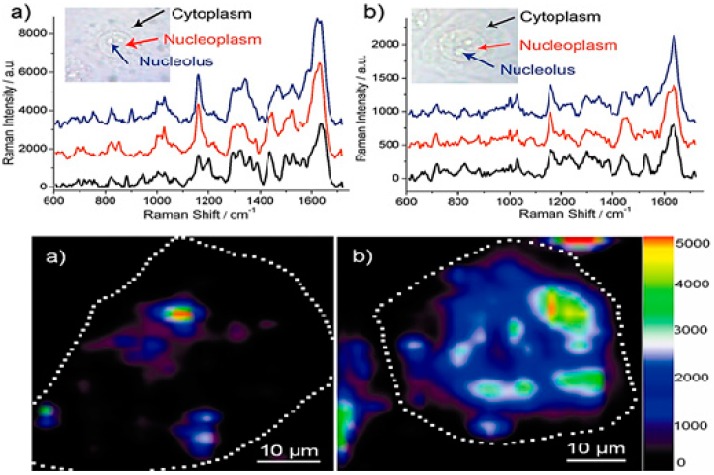
In the upper (**a**) GO/PVP/IGAuNPs and (**b**) IGAuNPs—SERS spectra of A549 cells collected from the regions corresponding to the cytoplasm, nucleoplasm, and nucleolus. In the lower—typical SERS images of A549 cells contained with (**a**) IGAuNPs or (**b**) GO/PVP/IGAuNPs, showing the distribution of gold nanostructures inside the cell. The dotted lines in the images are drawn to indicate the boundaries of select cells. Adapted from [220], with permission from ©2012 American Chemical Society.

**Figure 14:**
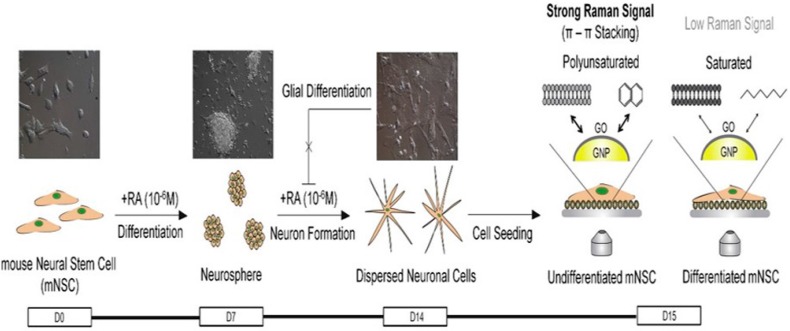
Schematic diagram representing the method to detect the undifferentiated and differentiated state of mNSCs using 3D GO-encapsulated AuNPs. Adapted from [139], with permission from ©2013 Elsevier.

Another group of scientists, reported a one-pot green technique for the intracellular synthesis of AuNS aided by poly(vinylpyrrolidone) (PVP)-functionalized GO [220]. The random intracellular distribution of GO/PVP/IGAuNPs in the cells allowed for ultrasensitive detection of cellular components of cancer cells (A549, 4T1, and HeLa cells) located in the cytoplasm, nucleoplasm, and nucleolus using SERS (Figure 13) and signals induced by the hybrid composites could be collected as early as 15 h, thus enabling the early detection or diagnosis of cancer as well. Specifically, a comparison of the SERS spectral analysis of GO/PVP/IGAuNPs and IGAuNPs individually showed that the hybrid structure results in five times larger Raman enhancement, possibly due to the formation of IGAuNP aggregates on GO [220]. More recently, Nergiz *et al.* [219] demonstrated a novel class of multifunctional hybrid nanopatches made up of GO and Au nanostars and the internalization of intact nanopatches into human epithelial breast cancer cells (SKBR-3) by Raman imaging. Raman mapping of the graphitic band of GO showed that hybrid nanopatches are concentrated in the cytoplasm with weak or no signal from the nucleus of the cell, thus indicating their presence in the cytoplasm and absence in the nucleus. In the cytosolic space, hybrid nanopatches exhibit long-term biocompatibility with extremely low cytotoxicity due to the amphipathic nature and large surface area of GO [219]. Kim and co-workers [139] moved towards the *in situ* monitoring of the undifferentiated or differentiated state or differentiation level of neural stem cells using 3D GO-encapsulated AuNPs by SERS. There is a positive correlation between the number of C=C bonds and the Raman intensity at 1656 cm^−1^. Indeed, the membranes of the undifferentiated cell line have polyunsaturated fatty acids which are richer in C=C bonds than normal/differentiated cells—this is the principle of differentiating cells by SERS (Figure 14).

**Figure 15:**
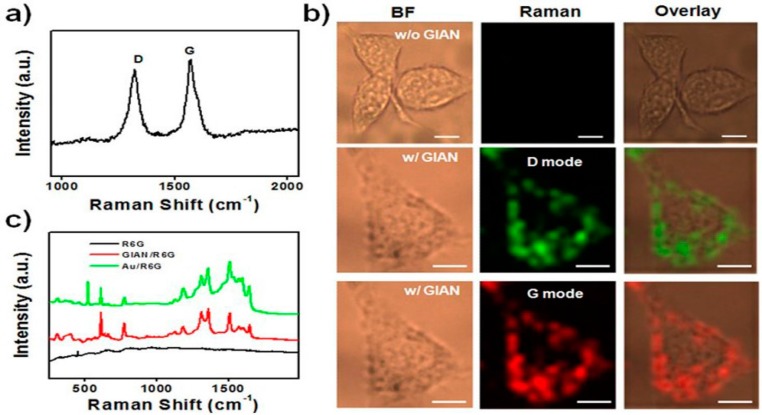
(**a**) Raman spectrum (excitation at 632 nm) of GIANs showing the G and D bands of graphitic carbon; (**b**) Raman imaging of MCF-7 cells with and without GIAN staining. BF: bright field, scale bar: 10 μm; (**c**) Raman spectra of R6G molecules, with and without GIAN, and with AuNPs, respectively. The figures are adapted from [138], with permission from ©2014 Nature Publishing Group.

Moreover, graphene–Au nanohexagons can differentiate between normal human breast cells, cancer cells, and cancer stem cells by Raman spectroscopy. These substrates in a concentration of 100 μg/1 × 10^4^ cells led to a 5.4-fold increase in the detection of breast cancer cells and 4.8-fold in sensitivity for the detection of breast cancer stem cells [207]. Bian *et al.* [138] fabricated GIAN nanostructures by employing the CVD process to wrap the AuNCs with a thin layer of graphene and verified it as a SERS substrate using the analyte R6G. GIANs significantly amplify the Raman signal by a factor of more than 100 by quenching background fluorescence and reducing the photocarbonization or photobleaching of analytes. These GIAN nanostructures are utilized for multimodal imaging of the breast cancer MCF-7 cells by significantly enhancing the Raman signals of the graphene shell by the AuNCs core while making the MCF-7 cells light up clearly under laser excitation (Figure 15). It is also evidently observed that the GIANs are distributed in the cytoplasm as the Raman signals are seen throughout the cytoplasm, not in the nuclei [138]. Moreover, an interesting and valued experiment is conducted by Wang *et al.* [223] using rGO–Au nanostars nanocomposites as active SERS materials for anticancer drug (doxorubicin) loading, its release thus showing its promising potential role in anticancer treatment for drug delivery of chemotherapeutic agents.

## 4. Conclusions, Challenges and Perspectives

Graphene–AuNPs hybrids display extraordinary synergistic properties when combined rather than individually. These materials have attracted considerable attention and been used in the biomedical and biosensing fields where biosensors are mainly based on electronic, electrochemical, and optical sensing principles. This review provides insights for graphene–AuNPs synthesis and discusses its importance and use in electrochemical and SERS biosensing platforms.

Synthesis techniques highly depend on the requirements of their intended applications. As such, synthesis methods of GO–AuNPs vary from green synthesis to synthetic, *in situ* (reduction, hydrothermal, electrochemical) to *ex situ* (covalent, noncovalent) AuNPs decoration, single layer to alternate LBL assembly, and even in some cases wrapping of AuNPs by GNs. It has been noted that graphene or rGO–AuNPs composites are used for electrochemical biosensor fabrication generally due to their greater conductivity and high electron mobility, hence *in situ* reduction is preferable in this case. Additionally, the *in situ* method is applicable whenever there is no urge for precise control over size, shape, and density of AuNPs and their narrow size distribution as well. Conversely, *ex situ* decoration is influenced by the predefined size, shape, and distribution of AuNS, which consequently minimizes possible incompatibilities between the synthesis and assembly of AuNPs on the graphene sheets. It has been noted that GO is generally used for the encapsulation of AuNS and their application has been successful in the stem cell differentiation biosensing by SERS. The review has shown that recent technological advancements in both graphene and AuNP synthesis are very promising to obtain high quality graphene with controllable size, shapes, layers, and defects in a cost-effective, high yielding, and ecologically friendly manner, in addition to AuNPs of the desired size, morphology, crystallinity, and good distribution on graphene sheets.

Graphene–AuNS have proven to be powerful sensing platforms for the fabrication of comparatively low cost, robust, rapid, and sensitive electrochemical biosensors. These electrochemical devices have been mostly applied in the biomedical fields for the detection of glucose either by enzymatic or catalytic means, H_2_O_2_, biomolecules (DNA, protein), small molecules (dopamine), microorganisms, food poisoning chemicals, environmental pollutants, and many more other analytes. One of the major achievements is the detection of a multi-drug resistant strain of *Mycobacterium tuberculosis* at fM level. All in all, electrochemical biosensors have the remarkable advantages of customization, miniaturization, and fast analysis times, however, they also have common analytical limitations, such as interferences from complex biological sample matrices and the inability to simultaneously detect multiple analytes at a time.

Most noticeably, the graphene–AuNPs SERS-based biosensor is trending in the scientific community due to its extremely high sensitivity as a result of its dual enhancement (chemical and electromagnetic). This dual effect facilitates larger enhancements as well as more clearly distinguished Raman peaks. This technology is being successfully utilized in multiple arenas for different purposes, most notably the detection of single molecular differentiation, multiplex DNA detection in a single laser excitation, screening of explosives, and security. One of the potential and exceptional applications is the bioimaging of intracellular components by SERS for the early detection of cancer cells, which may be a good alternative to conventional methods.

Graphene–AuNPs hybrid technology for biosensing is still in its infancy and, even though it is very promising, a few challenges are yet to be addressed. SERS bioimaging by the graphene–AuNPs hybrid has opened a new era but there needs to be extensive studies on long-term cytotoxicity, biocompatibility, and distribution of graphene–AuNPs hybrids *in vivo* for future applications. In summary, the design and manufacture of graphene–AuNPs hybrids and their implementation in biosensors is novel and very promising for sensing both *in vivo* and *ex vivo*. The technology permits the construction of highly sensitive, selective, customizable, and portable sensors for the detection of variable analytes. This is a promising sensing technology that can undoubtedly result in concrete innovations through concerted efforts between multidisciplinary teams which unite chemists, biochemists, material scientists, physicists, biologists, and engineers worldwide.

## Figures and Tables

**Table 1:** General properties of grapheme.

Properties	Value	References
Optical transmittance	~97.7%	[6]
Density	0.77 mg·m^−2^	[10,11]
Career density	10^12^ cm^−2^	[10,11]
Resistivity	10^−6^ Ω·cm	[10,11]
Planar surface area	2630 m^2^·g^−1^	[12]
Mechanical strength of its Young Modulus	1100 GPa	[13]
Fracture strength	125 GPa	[13]
Thermal conductivity	~5000 W·m^−1^·K^−1^	[14]
Mobility charge carrier	200,000 cm^2^·V^−1^·s^−1^	[15]

**Table 2:** Advantages and limitations of the major synthesis procedures of grapheme.

Synthesis Procedure	Beneficial Aspects	Limitations	References
Micromechanical exfoliation	Simple process. Few defects. Excellent quality of graphene. Well suited for fundamental research.	Poor reproducibility. Not amenable for large scale production.	[49,75,76]
CVD	Large area (up to ~1 cm^2^). Limited number of defects. Mass production. High quality graphene.	Expensive. Poor scalability.	[49,53,77,78]
Epitaxial growth	High quality of graphene. Few defects.	High cost. Requires high temp.	[49]
Colloidal suspension	Scalable. High volume of production. Suitable for multipurpose chemical functionalization.	Significant number of defects.	[79]
Unzipping of CNTs	Scalable with controlled widths and edge structures. Better control over chemical functionalization and edge quality.	Low yield. More expensive in respect to chemical exfoliation of graphite or graphite oxide.	[49,60]
Reduction of GO	Economical and facile technique.	Significant number of defects.	[76,79,80]

**Table 3:** Advantages and limitations of the major synthesis techniques of graphene–gold nanocomposites.

Synthesis Techniques	Advantages	Limitations	References
*In situ* reduction	One-pot synthesis. Efficient, easy to perform, and cost effective. Generally, no need of protecting surfactant or extra linker molecule. Can be employed by a lot of physical and chemical synthesis methods.	Hard to control the size and morphology of AuNPs in the resulting composite.	[25,83,84]
*Ex situ*	Prior synthesis of nanoparticles ensures good control over morphology, size, distribution and density of AuNPs on graphene.	Requires more time and steps.	[76,82,84]
Hydro-thermal	Synthesis of nanoparticles with high crystallinity and narrow size distribution. High production efficiency.	Requires high temp. and long reaction times, which may cause partial or complete reduction of GO.	[85,86,87]
Electro-chemical	Cost effective, robust and in most cases it’s a green approach. Morphology and size of the AuNPs can be fine-tuned by adjusting the electrodeposition potential, time and concentration of precursor solution.	Normally involves multiple steps.	[41,88,89,90,91,92]

**Table 4:** Summary of the different synthesis approaches of graphene–gold nanocomposites.

Name of the Synthesis Process	Name of the Final Graphene–Gold Hybrid	Gold—Functionalizing Agent (FA)/Stabilizing Agent (SA)/Reducing Agent (RA)	Graphene—Functionalizing Agent (FA)/Stabilizing Agent (SA)/Reducing Agent (RA)/Reduction Process (RP)	References
Seeded-growth simultaneous reduction	rGO-AuNPs	Sodium citrate (FA), NaBH_4_ (RA)	GO–rGO via redox chemistry of GO & Au Precursors (RP)	[111]
Sequential reduction method	N_2_ doped graphene-AuNPs	Ethylene glycol (RA)	Hydrazine hydrate & NH_3_ (RA)	[112]
Chemical reduction in micro flow reactor	GO-AuNPs	Dimethylamina borane (RA)	No agent	[113]
Eco-friendly chemical reduction method	rGO-AuNPs	Rose water (RA)	Rose water (RA)	[114]
Reductive deposition process	rGO-AuNPs	No agent	Hydrazine and NH_3_	[93]
Chemical reduction	Graphene-AuNPs	NaBH_4_ (RA) in presence of GO	Hydrazine hydrate (RA); SDS as a protector and disperser	[94]
Solution-based chemical reduction	Graphene-AuNPs	NaBH_4_ (RA)	ODA (FA)	[95]
Green synthesis method	GO-AuNPs	Tannic acid as RA and immobilizing agent	Tannic Acid (FA)	[97]
Electrostatic self- assembly	Graphene-AuNPs	NaBH_4_ (RA)	1-pyrene butyric acid (FA)	[96]
Seed-assisted reduction method	rGO-AuNPs	NaBH_4_ (RA) &Trisodium citrate (SA)	Pyrene ethylene glycol amine or decyl pyrene (FA)	[69]
Wet impregnation thermal reduction method	Graphene-AuNPs	A flow of H_2_/Ar (RA)	Hydrazine hydrate & NH_3_ (RA); Sulphur (FA)	[98]
Reduction via amidation reaction	GO-AuNPs	4 amino-thiophenol (FA)	Thionyl chloride (FA)	[83]
Chemical Reduction	Graphene-AuNPs	Sodium citrate (RA)	1050 °C for 30 s in furnace (RP); Hydrazine hydrate (RA)	[115]
Chemical reduction	GO-AuNPs	Sodium citrate (RA)	-	[81]
Green dual reduction method	rGO-AuNP	Ascorbic acid (RA)	Ascorbic acid (RA) Polyvinylpyrrolidone (SA)	[116]
One-pot green synthesis	GO-AuNPs	Tyrosine (RA)	No agent	[117]
Wet impregnation–thermal reduction method	GNs-AuNPs	Flow of H_2_/Ar (10% H_2_) by ramping temp. From room temp. to 350 °C (10 °C/min) and holding at 350 °C for 3 h	GO–GNs by Hydrazine hydrate and NH_3_	[98]
Microwave reduction	GO-AuNPs	Microwave exposure (1.05 kW, 2450 MHz)	No agent	[101]
Microwave-assisted simultaneous reduction	Graphene-AuNPs	Microwave exposure (0.8 kw) at 80 °C for 5 min under vigorous stirring	Hydrazine hydrate (RA)	[100]
Microwave irradiation—simultaneous reduction	Graphene-AuNPs	Microwave irradiation for 5 min	Ascorbic acid (RA)	[102]
Microwave-assisted simultaneous reduction	Graphene-AuNPs	Microwave exposure (0.2 kw) for 2 min	Polyethyleneimine (FA)	[103]
Sonolytic simultaneous and sequential reduction	Graphene-AuNPs	Ultrasonic frequency of 211 kHz	No agent	[105]
Sonochemical reduction	Graphene-AuNPs	Ultrasound irradiation	No agent	[104]
One-pot one step photochemical method	GO-AuNRs	UV-irradiation (256 nm, 30 W) for 25 min in a quartz tube	No agent	[106]
Photochemical reduction	Graphene-AuNDs	Photochemistry (RA) in presence of octadecanethiol	No agent	[107]
Photochemical reduction	Graphene-AuNS	Photo (LED) irradiation	No agent	[108]
Photo-assisted chemical reduction	Graphene-AuNPs	Laser light in presence of AuCl_4_^−^ electrolyte	No agent	[109]
Light-induced covalent interactions	rGO-AuNPs	3-aryl-3-(trifluoromethyl) diazirine (FA)	GO to rGO by high temp. (1050 °C for 30 s) reduction in an argon flow environment (RP)	[23]
Self-catalysis reduction	rGO-AuNPs	NaBH_4_ (RA) CTAB (SA)	NaBH_4_ (RA), GO–AuNPs (catalyst)	[84]
Self-assembly/Noncovalent attachment	rGO-AuNPs & GO-AuNPs	2-mercaptopyridine (FA), Trisodium citrate (SA/RA)	-	[82]
Thermal reduction of GO/electrostatic attractions	rGO-AuNPs	-	GO to rGO by thermal (200 °C) reduction in an argon flow environment	[96]
LBL self-assembly/electrostatic interactions	Graphene-AuNPs	Trisodium citrate (SA)	BSA (RA & SA)	[118]
-	Graphene-aerogel (GA)@AuNPs/AuNPs	Citric Acid (RA)	Ascorbic acid (RA) for GO to GA; freezing drying and thermal annealing at 180 °C for 6 h for final products	[119]

**Table 5:** *In situ* electrochemical reduction approaches for graphene–gold nanoparticles.

Electrode	Composition of Electrolytic Solution	Applied Electrochemical Potential and Reaction Condition	References
AuNPs/rGO/GCE	10 mM AuCl_3_, Nafion (0.5%), and 0.1 M H_2_SO_4_	−1.0 V for 500 s.	[41]
Au film/graphene–Au nanocomposite/GCE	0.1 mM HAuCl_4_	−1.2 V for 50 s for graphene; −0.25 V for 50 s for Au electrodeposition. Run the process using alternate graphene and AuNPs for 3 cycles.	[89]
DHB/AuNPs/rGO/GCE	0.3 mM HAuCl_4_	−0.2 V for 24 h at room temp.	[90]
Graphene/nano-Au/GCE	0.1 M Kn and 5 mM HAuCl_4_	−0.2 to 1.0 V for 2 cycles at scan rate of 50 mV∙s^−1^.	[91]
AuNP/electro reduced graphene (eGr)/Indium titanium oxide (ITO)	0.5 mM HAuCl_4_.nH_2_O in phosphate buffer	0 to −1.6 V continuously for 75 cycles at a scan rate 50 mV·s^−1^ to electrodeposit eGr on ITO and 25 cycles for AuNPs electrodeposition.	[120]
AuNPs/2,5-di-(2-thienyl)-1-pyrrole-1-(p-benzoic acid) (DPB)/graphene/Au electrode	3 mM of HAuCl_4_ containing 0.5 M H_2_SO_4_	−1.2 V for 200 s for electrochemical reduction and deposition of GO on Au electrode; −0.25 V for 25 s at 10 °C for the electrodeposition of AuNPs.	[121]
AuNPs/graphene-nanofibers/GCE	25 mM of HAuCl_4_ containing 0.1 M Na_2_SO_4_ solution	−0.4 V for 300 s for the electrochemical deposition of AuNPs.	[122]
AuNPs/graphene/GCE	mM HAuCl_4_ solution containing 0.5 M H_2_SO_4_	−1.2 V for 1200 s for the electrochemical reduction of GO on the electrode surface; −0.25 V for 30 s for the electrodeposition of AuNPs.	[123]
Graphene/nano-Au/GCE	0.1 M phosphate buffer (pH 7.0) containing 6.5 mM HAuCl_4_	0 to −2 V at a scan rate of 100 mV·s^−1^ for continuous cyclic voltammetric sweep of 40 cycles.	[124]
AuNPs/GO/GCE	HAuCl_4_ solution	Electrodeposition of AuNPs by pulse voltammetry with a pulse width of 0.1 s, potential 1.1 and −0.2 V, respectively.	[125]
AuNPs/ERGO/carbon ionic liquid electrode (CLIE)	5.0 mM HAuCl_4_ solution	−1.3 V for 300 s to from a stable ERGO on the surface of CILE; −0.4 V for 300 s for electrodeposition of AuNPs on ERGO/CILE.	[126]

**Table 6:** Graphene–gold nanocomposites-based electrochemical biosensors, target analytes with respective specificity of detection.

Composition of the Sensors	Detected Analyte	Linear Range of Detection	LOD	References
GOD/rGO–AuNPs/GCE	Glucose	1–8 mM	10 μM	[114]
Graphene/nano–Au/GOD/GCE	Glucose	0.2–2 and 2–20 mM	17 μM	[124]
Graphene/AuNPs/chitosan/GOD	Glucose	2–10 mM	180 μM	[159]
GOD/graphene–AuNPs/GCE	Glucose	0.1–10 mM	35 mM	[160]
Graphene Rod/AuNPs/GOD	Glucose	0.1–10 mM	83 μM	[162]
GCE–ATP–GNs–AuNPs–GOD	Glucose	1–12 mM (voltammetry)	9.3 μM	[98]
GCE–ATP–GNs–AuNPs–GOD	Glucose	1–8 mM (EIS)	4.1 μM	[98]
GOD/graphene–AuNPs	Glucose	0.02–2.26	4.1 μM	[163]
GA@AuNPs/AuNPs	Glucose	0.01–16 mM	4.0 μM	[119]
Graphite/SrPdO_3_/AuNPs	Glucose	0.1–6 mM	10.1 μM	[164]
GOD–graphene/PANI/AuNPs/GCE	Glucose	0.004–1.12 mM	0.6 μM	[161]
Graphene/Au–NPs/GCE	H_2_O_2_	0.0005–0.5 mM	0.44 μM	[165]
Nafion/Hb/AuNPs–graphene/GCE	H_2_O_2_	0.0001–0.07 mM	0.03 μM	[87]
GCE–GO–AuNP–ssDNA	DNA	-	100 fM	[125]
ssDNA/AuNPs–ATPGO/GCE	DNA	1.0 × 10^−13^ to 1.0 × 10^−9^ M	1.13 × 10^−14^ M	[173]
ssDNA/AuNPs/TB–GO/GCE	MDR gene (DNA)	1.0 × 10^−11^ to 1.0 × 10^−9^ M	2.95 × 10^−12^ M	[168]
Capture probe (cDNA)/AuNPs–rGO/GCE	DNA	0.1 μM to 0.1 fM	35 aM	[167]
Au film/graphene–Au nanocomposite/GCE	Peanut allergen Ara h1 gene	10^−16^ to 10^−13^ M	0.041 fM	[89]
DHB/AuNPs/rGO/GCE	levodopa (LD)	0.05–1200.0 μM	18 nM	[90]
Au NP/GO/GCEs	Ascorbic Acid	0.11–0.6 mM	100 nM	[81]
Graphene/AuNPs/GCE	DES	1.20 × 10^−8^ to 1.20 × 10^−5^ M	9.80 × 10^−9^ M	[91]
AuNPs/rGO/GCE	NADH in human urine	50 nM to 500 μM	1.13 nM	[41]
AuNPs/ERGO/CILE	Folic Acid	0.01 μM to 50.0 μM	2.7 nM	[126]
AuNPs/1-pyrene butyric acid-functionalized graphene/GCE	Uric acid	2.6 × 10^−6^ to 6.2 × 10^−5^ M	2.0 × 10^−7^ M	[96]
Graphene nanosheet–PEI/AuNPs/GCE	Dopamine	2.0 to 48.0 μM	0.2 μM	[103]
[AuNPs/rGO]_20_/GCE	Dopamine	1.0 to 60.0 μM	0.02 μM	[135]
Hb/AuNPs–graphene–SDS/BPG	Nitric oxide	7.2 × 10^−7^ to 7.92 × 10^−6^ M	1.2 × 10^−8^ M	[94]
Hb/AuNPs/GACS/GRE	Nitrite	0.05 to 1000 μM	0.01 μM	[172]
AuNPs/graphene nanofibers/GCE	Bisphenol A in baby bottle	8.0 × 10^−8^ to 2.5 × 10^−4^ M	3.5 × 10^−8^ M	[122]
Anti-BPA/MCH/AuNPs/graphene/GCE	Bisphenol A in milk sample	0.01–10.0 μM	5 nM	[123]
Aflatoxin B_1_ antibody-AuNPs/DPB/graphene/Au electrode	Aflatoxin B_1_ in spiked food	3.2 fM–0.32 pM	1 fM	[121]
AuNP/N_2_-doped graphene/GCE	Chloramphenicol	2.0 × 10^−6^ to 8.0 × 10^−5^ M	5.9 × 10^−7^ M	[112]
Anti-estradiol antibody-AuNP–eGr/ITO	17 β-estradiol	1 × 10^−3^ to 0.1 × 10^−12^ M	0.1 fM	[120]
rGO–AuNPs-modified GCE	*M. tuberculosis*	1.0 × 10^−15^ and 1.0 × 10^−9^ M	fM level	[169]
*E. coli* O157:H7 antibodies-AuNPs/rGOPE	*E. coli* O157:H7	1.5 × 10^2^ to 1.5 × 10^7^ cfu/mL	1.5 × 10^2^ cfu/mL	[170]
Au-graphene–AuNPs electrode	Carbamazepine	5 × 10^−6^ to 10^−2^ M	3.03 × 10^−6^ M	[110]
anti-CEA/AuNPs/Arg/rGO/CILE	CEA	0.5 to 200 ng·mL^−1^	0.03 ng·mL^−1^	[171]
AuNP–graphene/CILE	Hydroquinone	0.06 μM to 800.0 μM	0.018 μM	[100]

**Table 7:** SERS enhancement of the graphene–gold hybrid nanocomposites.

Name of the Hybrid Substrate	SERS—Order of Magnitude	Compared Material	References
rGO–AuNPs	100	Pure AuNPs	[42]
Graphene–AuNS	45	Graphene	[43]
Graphene–AgNS	150	Graphene	[43]
AuNPs/graphene/SiO_2_/Si	120	Graphene/SiO_2_/Si	[199]
Graphene–AuNPs	10–100	AuNPs	[212]
Graphene–AuNPs	77.6	Graphene	[213]
Pyrene ethylene glycol amine-functionalized rGO/AuNRs	14.7	Bare rGO	[69]
R6G/GO–AuNR with CTAB	10	Pure AuNRs	[200]
Nano GO (nGO)–Au nanostars	5.3	nGO	[214]
Graphene–AuNPs	3.3	AuNPs	[215]
R6G/AuNP/graphene/SiO_2_/Si	86	Graphene/SiO_2_/Si with R6G	[216]
Ag/rGO/Au for rhodamine B (RhB)	8.8	Pristine Ag dendrites	[217]
AuNPs/rGO/SiO_2_/Si	40	Blank substrate	[209]
GO–AuNPs	~4	GO	[218]
Neural Stem Cells on GO encapsulated AuNPs	3.5	AuNPs	[139]
GO–AuNS	3	GO	[219]
GO/PVP/intracellularly grown AuNPs (IGAuNPs)	5	IGAuNPs	[220]
Carbaryl on graphene–Au film–AuNR	2	Au film–AuNR	[202]
Carbaryl on graphene–Au film–AuNR	100	Graphene–AuNR	[202]
Si/N_2_ doped diamond-like carbon (DLC-N)/Au/rGO/Au for RhB	860	Si/DLC-N	[221]
GO-popcorn shaped AuNPs hybrid for R6G	11	GO	[203]
GO-Au nanocage for RDX	4	Au nanocage	[211]
Graphene-isolated AuNC (GIAN) nanostructures for R6G	More than 100	R6G	[138]
Au@AgNPs/GO/Au@AgNPs sandwich for R6G	Enhancement factor of ~7.0 × 10^7^	-	[193]

## Abbreviations

APTES	Aminopropyltriethoxysilane
ATP	4-aminothiphenol
Arg	Arginine
Ar	Argon
CBZ	Carbamazepine
CILE	Carbon Ionic Liquid Electrode
CNT	Carbon Nanotube
CEA	Carcinoembryonic Antigen
CTAB	Cetyl-trimethylammonium bromide
CR-GO	Chemically Reduced Graphene Oxide
CVD	Chemical Vapor Deposition
cfu	Colony Forming Unit
RDX	Cyclotrimethylenetrinitramine
Cy3	Cysteamine
DES	Diethylstilboestrol
DHB	Dihydroxybenzoic Acid
EIS	Electrochemical Impedance Spectroscopy
EPA	Environmental Protection Agency
fM	Femtomolar
FAO	Food and Agriculture Organization
FA	Functionalizing Agent
GCE	Glassy Carbon Electrode
GOD	Glucose Oxidase
AuNC	Gold Nanocrystal
AuNP	Gold Nanoparticle
AuNS	Gold Nanostructure
GA	Graphene Aerogel
GNs	Graphene Nanosheets
GO	Graphene Oxide
Hb	Hemoglobin
H_2_O_2_	Hydrogen Peroxide
ITO	Indium Titanium Oxide
kW	Kilowatt
LBL	Layer by layer
LOD	Limit of Detection
MHz	Megahertz
MBA	Mercaptophenyl Boronic Acid
MPTMS	3-mercaptopropyltrimethoxysilane
MRSA	Methicillin Resistant *Staphylococcus aureus*
μM	Micromolar
mM	Milimolar
MDR	Multidrug Resistant
NDs	Nanodots
nM	Nanomolar
nGO	Nano graphene oxide
NR	Nano Rod
NADH	Nicotinamide Adenine Dinucleotide
N_2_	Nitrogen
ODT	Octadecanethiol
ODA	Octadecylamine
O_2_	Oxygen
ppm	Parts per million
PEG	Pegylated
pM	Picomolar
Pt	Platinum
PANI	Polyaniline
PAH	Poly allylamine hydrochloride
PLA	Poly (lactic acid)
PVP	Poly(vinylpyrrolidone)
KClsat	Potassium Chloride saturated
rGO	Reduced Graphene Oxide
RA	Reducing Agent
RP	Reducing Process
Ref.	Reference
SCE	Saturated Calomel Electrode
SEM	Scanning Electron Microscopy
Si	Silica
SiO_2_	Silica Oxide
Ag	Silver
AgCl	Silver Chloride
AgNS	Silver nanostructures
ss	Single Stranded
SDS	Sodium Dodecyl Sulfate
SA	Stabilizing Agent
SERS	Surface Enhanced Raman Spectroscopy/Scattering
TR-GO	Thermally Reduced Graphene Oxide
3D	Three-dimensional
TB	Toluidine Blue
TEM	Transmission Electron Microscopy
2D	Two dimensional
wt	Weight
WHO	World Health Organization
